# A cause of recurrent strokes: carotid webs detected by CT angiogram

**DOI:** 10.1259/bjrcr.20170066

**Published:** 2017-11-09

**Authors:** Hannah Smyth, Danielle Byrne, Derek Hayden, Eoin Kavanagh, Sean Murphy

**Affiliations:** ^1^ Department of Stroke Medicine, Mater Misericordiae University Hospital, Dublin, Ireland; ^2^ Department of Radiology, Mater Misericordiae University Hospital, Dublin, Ireland

## Abstract

Carotid webs are an uncommon cause of recurrent ischaemic strokes. They are considered a form of fibromuscular dysplasia, possibly developmental in origin, with non-inflammatory and non-atherosclerotic features and a characteristic appearance on CT angiography. They have been described as a thin intraluminal filling defect along the posterior wall of the carotid bulb in oblique sagittal reformats and a septum on axial CT angiography. Here we summarize two cases of ischaemic strokes secondary to carotid webs with characteristic images. Detection and awareness of carotid webs and their imaging features among radiologists and physicians are important as it is associated with a high risk of recurrent cerebrovascular events.

## Case 1

**Figure 1.**
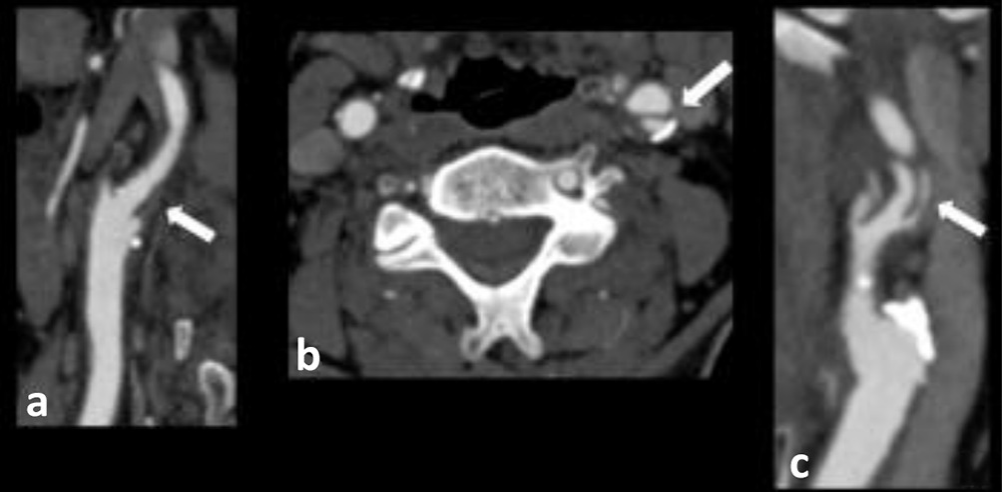
85-year-old male patient who presented with acute right-sided weakness. (a) Sagittal, (b) axial and (c) coronal CTA images demonstrate a carotid web arising from the posterior wall of the proximal left ICA. CTA, CT angiogram; ICA, internal carotid artery.

**Figure 2.**
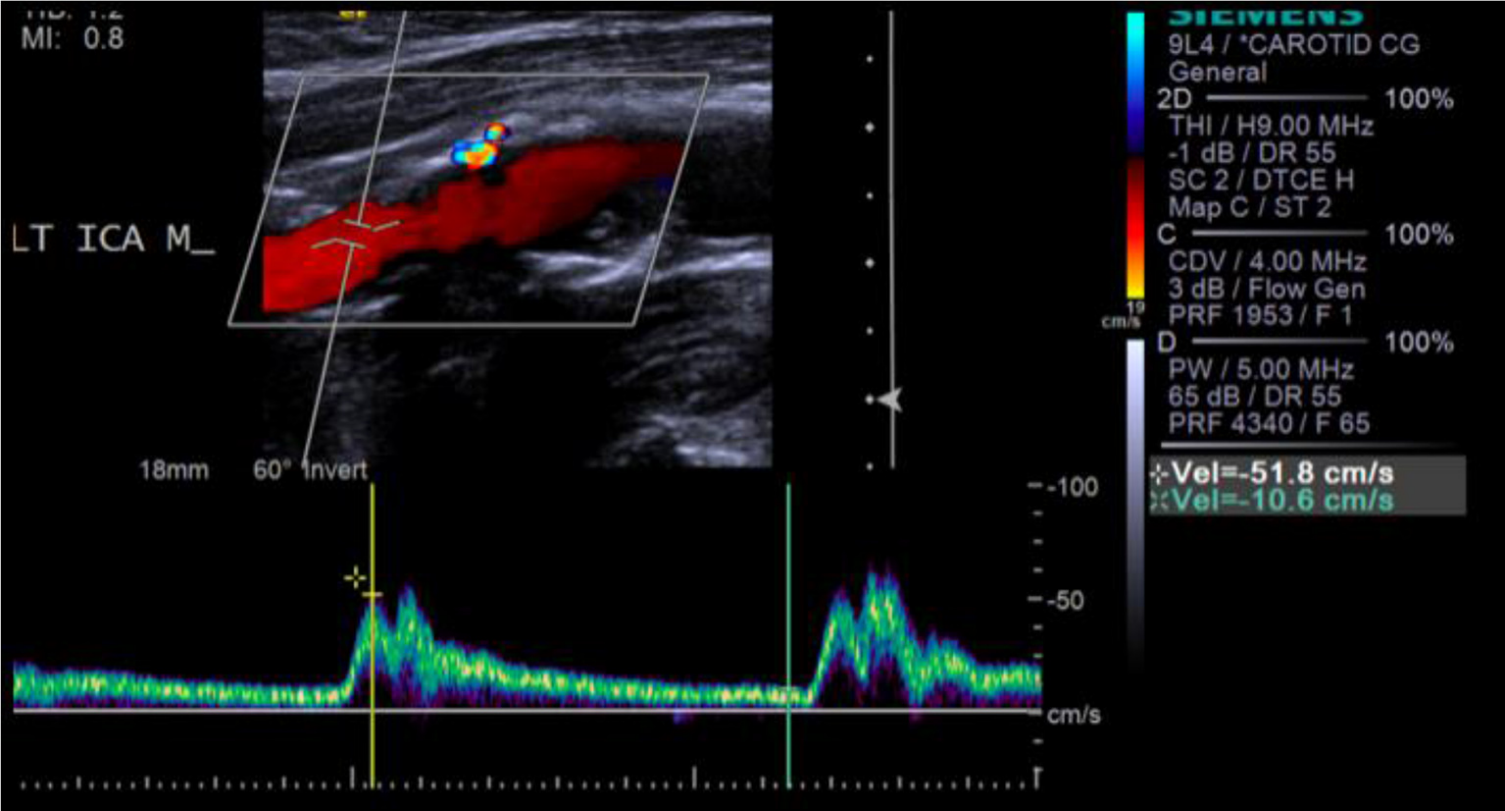
85-year-old male patient who presented with acute right-sided weakness. Carotid duplex ultrasonography left ICA: irregular echogenic plaque at the origin of the left ICA resulting in 0–29% stenosis. ICA, internal carotid artery.

**Figure 3.**
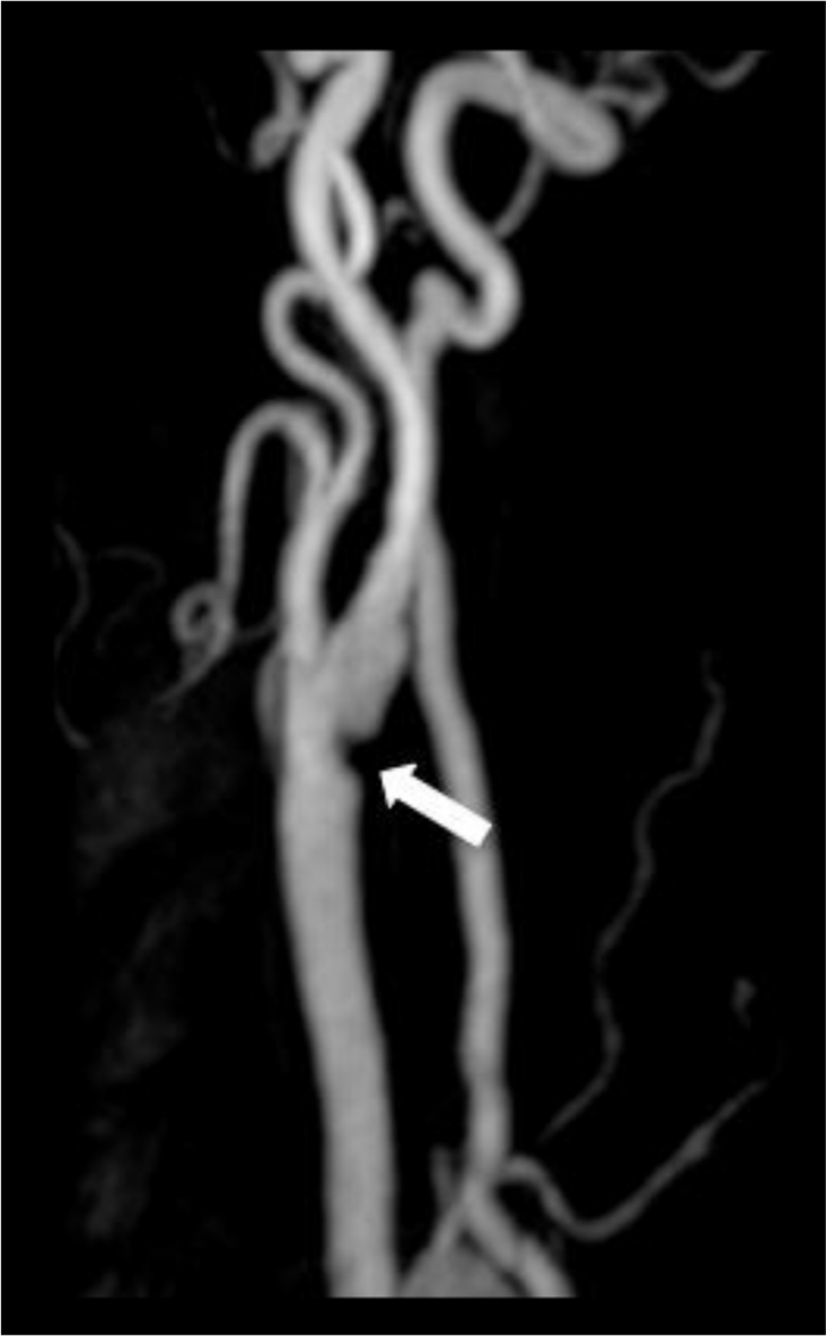
85-year-old male patient who presented with acute right-sided weakness. Contrast enhanced MRA of the left carotid demonstrates a web arising from the proximal left ICA at the level of the carotid bifurcation. ICA, internal carotid artery; MRA, magnetic resonance angiogram.

An 85-year-old right-handed male presented with sudden onset right hemiparesis. His medical history was significant for hypertension, dyslipidaemia and prostate cancer. He never smoked cigarettes and had no known diabetes mellitus or atrial fibrillation. Pre-admission cardiovascular medications included aspirin 75 mg and atorvastatin 20 mg daily. Admission National Institutes of Health Stroke Scale was 6. Non-contrast CT head revealed established bilateral corona radiata infarcts. CT angiogram (CTA) demonstrated a left internal carotid artery (ICA) web (Figure 1). Intravenous thrombolysis was administered with a door to needle time of 21 min. On carotid duplex ultrasonography, an irregular mixed echogenic plaque (query ulcerated) was seen at the origin of the left ICA causing a 0–29% stenosis (Figure 2). MRI head demonstrated acute infarcts in the left parietal lobe. Magnetic resonance angiogram (MRA) carotid arteries also demonstrated a shelf-like projection arising from the posterior wall of the left ICA consistent with a carotid web (Figure 3).

For secondary stroke prevention, he was prescribed clopidogrel 75 mg daily and atorvastatin 40 mg. He was subsequently transferred to a stroke rehabilitation centre.

## Case 2

A 38-year-old Middle Eastern male presented to the emergency department with sudden onset dense right hemiparesis, right facial droop and aphasia. His background history included a previous ischaemic stroke 15 months ago treated in a different institution. At that time, he presented with a headache and dysphasia and a CT brain showed a left temporo-parietal infarct. By 6 months later, he had returned to his baseline of full functional independence on treatment with clopidogrel 75 mg once daily and atorvastatin 40 mg daily.

On this admission, CT brain showed a left M1 occlusion and the patient was treated with intravenous alteplase and thrombectomy.

**Figure 4.**
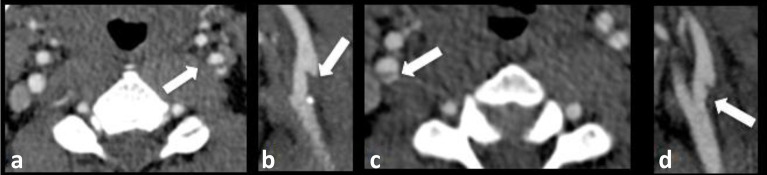
Bilateral carotid webs. CTA images of a 39-year-old male patient who presented with an acute left M1 occlusion with a background history of a prior left MCA infarct. (a and b) Axial and sagittal CTA images demonstrate a carotid web arising from the posterior wall of the proximal left ICA. (c and d) Axial and sagittal CTA images demonstrate a carotid web arising from the posterior wall of the proximal right ICA. CTA, CT angiogram; ICA, internal carotid artery; MCA, middle cerebral artery.

**Figure 5.**
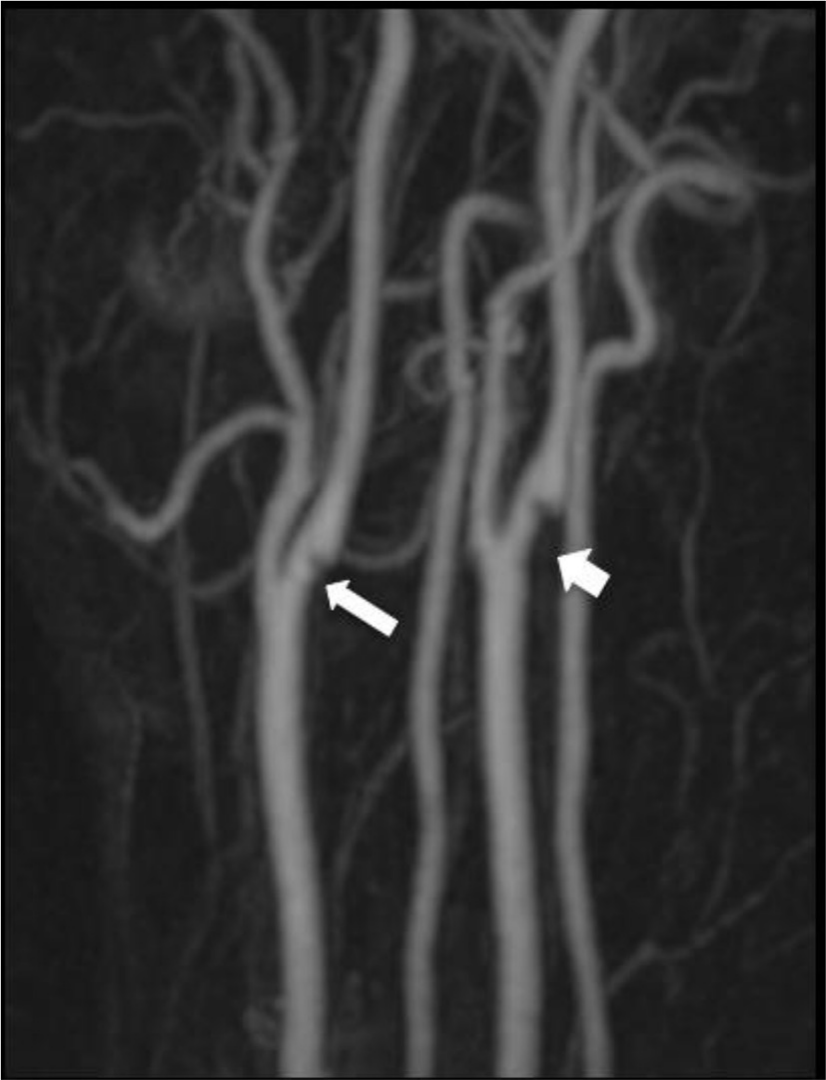
39-year-old male patient who presented with an acute left M1 occlusion with a background history of a prior left MCA infarct. Contrast enhanced MRA MIP image of the neck arteries demonstrate webs arising from the posterior walls of the proximal right (long arrow) and left (short arrow) internal carotid arteries. MCA, middle cerebral artery; MRA, magnetic resonance angiogram.

**Figure 6.**
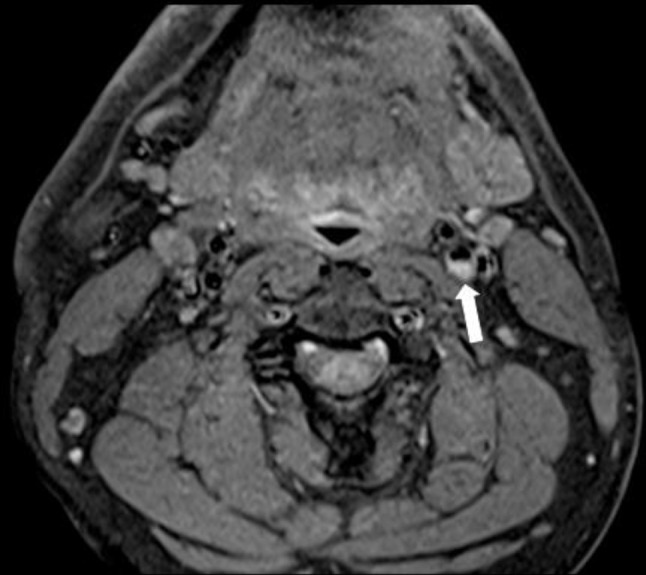
39-year-old male patient who presented with an acute left M1 occlusion with a background history of a prior left MCA infarct. Axial fat-saturated T1W MRI: crescentic hyper intense signal at the posterior aspect of the origin of the left ICA consistent with haemorrhage within the known carotid web. ICA, internal carotid artery; MCA, middle cerebral artery; T1W, *T*_1_ weighted.

His CT angiogram intracranial confirmed an acute occlusion of the M1 portion of the left middle cerebral artery and revealed two separate foci of soft plaques arising from the posterior wall of the origin of the left and right ICA with accompanying carotid webs on both sides (Figure 4). His MRA carotids showed a haemorrhagic “plaque” at the origin of the left ICA but no high-grade ICA stenosis or any evidence of dissection (Figure 5). Axial fat-saturated T1W MRI demonstrated a crescentic hyperintense signal at the posterior aspect of the origin of the left ICA consistent with haemorrhage within the known carotid web (Figure 6). His carotid Doppler was normal. Extensive stroke work-up did not reveal any other cause for his stroke.

He was started on dabigatran 150 mg twice daily and aspirin 75 mg. He has now been transferred to a stroke rehabilitation centre with residual speech apraxia, improving right limb weakness and ability to comprehend one step commands.

## Discussion

These cases describe two males who presented with ischaemic strokes due to carotid webs. Carotid webs are an exceptionally rare cause of ischaemic stroke. Previous literature has described the lesions as a congenital shelf or web-like projection into the lumen of the proximal internal carotid artery near the carotid bulb.^1,2^ They are considered a form of fibromuscular dysplasia, possibly developmental in origin, with non-inflammatory and non-atherosclerotic features.^2,3^ In cases of FMD the affected vessel typically shows a “string of beads” appearance on vascular imaging.^4^ In contrast, a carotid web appears as a small, shelf-like linear filling defect projecting superiorly into the arterial lumen and arising from the posterior wall of the proximal internal carotid artery^5^ on all imaging modalities. The differential diagnosis of a carotid web includes an atherosclerotic plaque, dissection, post-traumatic aneurysm or thrombus.^6^

On ultrasound, a carotid web appears as a linear echogenic filling defect, arising from the posterior wall of the origin of the internal carotid artery (Figure 2). Diagnosis of a carotid web with ultrasound can be difficult due to its small size, non-flow limiting nature^5,7^ and lack of awareness of this entity among sonographers.^6^ An atherosclerotic plaque appears as a focal eccentric plaque with or without intimal calcifications on ultrasound and a plaque-like filling defect typically located at the arterial bifurcation on CTA.^6^ One of the features associated with recent plaque rupture or ischaemic stroke is plaque ulceration,^8^ which is characterized as an indentation, fissure or erosion on the luminal surface of a plaque^9^ resulting in exposure of the necrotic core of the plaque to the circulation.^8,10^ Plaque ulceration can be identified on ultrasound, CTA and MRA^11^ and is a feature that can differentiate a carotid plaque from a web. In some cases, it is difficult to distinguish a carotid web from an atherosclerotic plaque which may appear web-like,^12^ but the former should be suspected in patients with no cardiovascular risk factors and recurrent “cryptogenic” strokes.^13^

Ultrasound imaging features for a carotid artery dissection include an intramural haematoma with expansion of the vessel calibre and intimal flap within the vessel lumen, however ultrasound evaluation is limited, as the entire vascular axis cannot be assessed.^14^ A dissection flap, which propagates beyond the bulb with a true and false lumen, can be identified on CTA.^6^ MRI is the standard non-invasive investigation of choice in cases of suspected carotid artery dissection allowing evaluation of the vascular lumen with MR angiography for evidence of an intimal flap, stenosis, pseudo aneurysm formation or occlusion, identification of mural haematoma on T1 fat-suppressed imaging and cerebral ischaemia on diffusion weighted imaging.^14^ Thus, cross-sectional imaging with CTA and MRI will allow definitive differentiation between a carotid web and dissection.

Ultrasound can be used to identify a carotid artery pseudo aneurysm with duplex Doppler flow demonstrating the characteristic “to and fro” waveform^15^ which is not seen in the setting of a carotid web while CTA and MR are also adequate non-invasive imaging modalities in the assessment of pseudoaneurysms.^16^

Detection and awareness of a carotid web is important as it is associated with a high risk of recurrent cerebrovascular events. In a previous paper, recurrent stroke was seen in 71.4% of patients with a carotid web.^9^ Carotid webs can cause ischaemic strokes either due to significant carotid stenosis or by facilitating the formation of thrombus within a cavity between the web itself and posterior wall of the ICA, with subsequent distal embolization as suggested by Choi et al.^9^

Previous literature has emphasized the superiority of conventional digital subtracted angiography over non-invasive imaging to detect carotid webs.^17^ However, in our cases, CTA identified the typical appearance of carotid webs and the patients did not undergo DSA which had previously been recommended to detect carotid webs^17^ due to greater contrast and spatial resolution enabling dynamic real time vascular evaluation.^6^ CTA has more recently been demonstrated to be accurate for the identification and characterization of carotid web.^6,9^ MRI may provide additional information for characterization of the nature of carotid webs.

The MRI in case 2 suggested a haemorrhagic plaque at the origin of the left internal carotid artery with no high-grade internal carotid artery stenosis (Figure 6). Similar to this case, MRI has previously been reported to be useful for the identification of haemorrhage within a carotid web, which on CTA may indistinguishable from haemorrhage within an atherosclerotic plaque.^18^ It remains to be determined whether haemorrhage within a carotid web renders a patient at increased risk of embolic ischaemic events compared with a non-haemorrhagic carotid web. CTA has the advantage of rapidly generating high-resolution imaging in multiple planes with characterization of additional findings such as thrombus^13^ while MRA takes longer to perform and is generally less widely available than CT.^19^

Optimal management of carotid webs remains uncertain and exclusively based on previous case reports. Interventional management options described to date have included carotid stenting, carotid endarterectomy, surgical excision or balloon dilatation with previous good clinical results reported.^3,20^ Medical management includes secondary prevention with aspirin and optimal medical management of cardiovascular risk factors. In our second reported case, our patient suffered a second embolic stroke despite anti-platelet therapy with clopidrogrel. This, plus the fact that the web seemed to contain haemorrhagic material, led us to prescribe empirical additional anticoagulant therapy. Antiplatelet therapy alone seems insufficient to prevent stroke recurrence as judged by previously reported cases of stroke recurrence despite treatment with aspirin and optimization of cardiovascular risk factors.^21^

Our choice of dabigatran for secondary stroke prevention has not been previously reported and its use is based upon desire to add an anticoagulant drug with lower risk of intracranial bleeding than a vitamin K antagonist coupled with achieving a consistent degree of therapeutic anticoagulation in a 35-year-old male with recurrent strokes. Our second case also has bilateral carotid webs, one of which is asymptomatic and the frequency of bilateral webs is to date unreported and conservative medicine therapy has been indicated for asymptomatic cases.^2^

To date, there has been very limited long-term follow up of patients with carotid webs and therefore little is known about the long-term clinical success of medical or interventional treatment options.

## Learning points

1. Carotid webs are an uncommon cause of recurrent ischaemic strokes which may lead to severe disability and morbidity.
2. Heightened awareness and knowledge of carotid webs and their imaging features among physicians and radiologists is needed to avoid overlooking this important cause of recurrent stroke.
3. They have a characteristic appearance on non-invasive vascular imaging (CT angiography) which can be mistaken for carotid atherosclerosis. Typical findings of carotid web on non-invasive vascular imaging means that invasive digital subtraction angiography is not necessary to confirm the diagnosis.
4. There is lack of consensus on most appropriate treatment which ranges from conservative treatment of asymptomatic cases to surgical intervention (*e.g*. stenting, carotid endarterectomy). Aggressive secondary preventive therapy with anti-thrombotic therapy is justified given the high risk of recurrent strokes association with carotid webs.

## Consent

Written informed consent for the case to be published (including images, case history and data) was obtained from the patient(s) for publication of this case report, including accompanying images.
